# The oxygen dilemma: The challenge of the anode reaction for microbial electrosynthesis from CO_2_

**DOI:** 10.3389/fmicb.2022.947550

**Published:** 2022-08-03

**Authors:** Maliheh Abdollahi, Sara Al Sbei, Miriam A. Rosenbaum, Falk Harnisch

**Affiliations:** ^1^Department of Environmental Microbiology, UFZ-Helmholtz Centre for Environmental Research, Leipzig, Germany; ^2^Bio Pilot Plant, Leibniz Institute for Natural Product Research and Infection Biology - Hans-Knöll Institute, Jena, Germany; ^3^Faculty of Biological Sciences, Friedrich Schiller University Jena, Jena, Germany

**Keywords:** carbon dioxide valorization, microbial electrosynthesis, microbial electron uptake, extracellular electron transfer, oxygen stress

## Abstract

Microbial electrosynthesis (MES) from CO_2_ provides chemicals and fuels by driving the metabolism of microorganisms with electrons from cathodes in bioelectrochemical systems. These microorganisms are usually strictly anaerobic. At the same time, the anode reaction of bioelectrochemical systems is almost exclusively water splitting through the oxygen evolution reaction (OER). This creates a dilemma for MES development and engineering. Oxygen penetration to the cathode has to be excluded to avoid toxicity and efficiency losses while assuring low resistance. We show that this dilemma derives a strong need to identify novel reactor designs when using the OER as an anode reaction or to fully replace OER with alternative oxidation reactions.

## Introduction

Microbial electrochemical synthesis (MES) is the execution of microbially catalyzed electrochemical reactions to transform a substance into the desired product (Schröder et al., 2015). In simple words, MES is the generation of valuable extracellular multicarbon materials using electric energy based on combining microbial and electrochemical transformations (Zhang et al., 2013). MES can be based on anodic as well as cathodic reactions and covers the production of a variety of chemicals (refer to also Table 1) (Schröder et al., 2015). One highly interesting carbon substrate for cathodic MES is carbon dioxide (CO_2_).

**TABLE 1:** Collection examples of cathodic microbial electrosynthesis studies using CO_2_ substrate.

Microorganisms	Product of cathodic MES	Cathode material	Anode material	Anode reaction	BES design/ use of membrane	References
Mixed culture	Acetate	NanoWeb-Reticulated Vitreous Carbon (RVC) & unmodified RVC	Platinum wire	2H_2_O → O_2_ + 4H ^+^+ 4e^–^	Two chamber/ CEM Cationic Exchange Ultrex CM17000, Membranes International	Jourdin et al., 2014
*Sporomusa ovata*	Acetate, 2-oxobutyrate.	Unpolished graphite rods	Unpolished graphite rod	2H_2_O → O_2_ + 4H ^+^+ 4e^–^	Two chamber/ Nafion CEM (No specification)	Nevin et al., 2010
Enriched brewery WW sludge	Acetate	Graphite granules	Graphite rod under graphite granules	2H_2_O → O_2_ + 4H ^+^+ 4e^–^	Two chamber/ Nafion 117	Marshall et al., 2013
Anaerobic digester/ Retention basin	Acetate	Granular graphite and graphite rods	Granular graphite and graphite rods	2H_2_O → O_2_ + 4H ^+^+ 4e^–^	Two chamber/ CEM CEM (CMI-7000, Membranes International)	Batlle-Vilanova et al., 2016
*Clostridium aceticum* *Sporomusa sphaeroides* *Clostridium ljungdahlii*, *Moorella thermoacetica*	Acetate, 2-oxobutyrate Acetate Acetate Acetate	Graphite rod	Graphite rod	2H_2_O → O_2_ + 4H ^+^+ 4e^–^	Two chamber/ Nafion 117	Nevin et al., 2011
*S. ovata*	Acetate	Graphite rods	Graphite rod	2H_2_O → O_2_ + 4H ^+^+ 4e^–^	One chamber/ Membrane-less reactor, with anode on the top	Giddings et al., 2015
Enriched acetogenic culture	Acetate	RVC foam	Mixed metal oxide (IrO_2_/Ta_2_O_5_)	2H_2_O → O_2_ + 4H ^+^+ 4e^–^	Two chamber/ CEM (CMI-7000, Membranes International)	LaBelle and May, 2017
Enriched mixed culture	Acetate, *n*-butyrate, *n*-caporate	Three carbon felts stacked together	Pt/IrO_2_ coated Ti	2H_2_O → O_2_ + 4H ^+^+ 4e^–^	Two chamber/ CMI-7000,	Jourdin et al., 2018
Enriched mixed culture	Acetate, Butyrate, Caproate	Graphite granules, Carbon felts stacked together	Pt/IrO_2_ coated Ti	2H_2_O → O_2_ + 4H ^+^+ 4e^–^	Two chambers/ CEM (Fumasep FKS, Fumatech BWT)	Jourdin et al., 2019
Enriched mixed culture	Acetate, carboxylic acids and Ethanol	Carbon cloth (CC) and stainless steel mesh (SS), and CC-SS with activated carbon (AC)	Plain graphite plate	Wastewater + Glucose → Treated wastewater + CO_2_	Two chamber/ CEM (No Specification)	Annie Modestra and Venkata Mohan, 2019
Raw + acclimated activated sludge	Capraoate, volatile fatty acids	Carbon felt	Ti mesh coated with Ir and Ru	2H_2_O → O_2_ + 4H ^+^+ 4e^–^	Two chamber/ CEM (Shanghua Water Treatment Materials Co. Ltd., Shanghai, China)	Jiang et al., 2020
*S. ovata*	Acetate	Chitosan on carbon cloth	Graphite rod	2H_2_O → O_2_ + 4H ^+^+ 4e^–^	Two chamber/ Nafion 117	Zhang et al., 2013
Enriched mixed culture	Acetate	3D RVC with multi-walled carbon nano-tubes (MWCNT)	Platinum wire	2H_2_O → O_2_ + 4H ^+^+ 4e^–^	Two chamber/ Ultrex CM17000,	Jourdin et al., 2015
Enriched mixed culture	Acetate	MWCNT-RVC	Platinum wire	2H_2_O → O_2_ + 4H ^+^+ 4e^–^	Two chamber/ Ultrex CM17000	Jourdin et al., 2016
*S. ovata* adapted on Methanol	Acetate	CC-rGO-TEPA	Graphite rod	2H_2_O → O_2_ + 4H ^+^+ 4e^–^	Two chamber/ Nafion 115	Chen et al., 2016
*S. ovata*	Acetate	3D-Graphene carbon felt composite	Graphite rod	2H_2_O → O_2_ + 4H ^+^+ 4e^–^	Two chamber/ Nafion 115	Aryal et al., 2016
*S. ovata*	Acetate	Graphene paper	Graphite rod	2H_2_O → O_2_ + 4H ^+^+ 4e^–^	Two chamber/ Nafion 115	Aryal et al., 2017
*S. ovata*	Acetate	Carbon cloth coated with poly(3,4 ethylenedioxythiophene):polystyrene sulfonate (PEDOT:PSS)	Graphite rod	2H_2_O → O_2_ + 4H ^+^+ 4e^–^	Two chamber/ Nafion 115	Aryal et al., 2018
*S. ovata*	Acetate	Graphite rod -Ni Nano wire	Graphite rod	2H_2_O → O_2_ + 4H ^+^+ 4e^–^	Two chamber/ Nafion 117	Nie et al., 2013
*S. ovata*	Acetate	3D Iron oxide modified carbon felt	Graphite rod	2H_2_O → O_2_ + 4H ^+^+ 4e^–^	Two chamber/ Nafion 117	Cui et al., 2017
Mix culture from MFC	Acetate, *n*-butyrate, *iso*-butyrate, n-caproate, volatile fatty acids and their alcohols	3D Graphene Ni-foam	Pt wire	2H_2_O → O_2_ + 4H ^+^+ 4e^–^	Two chamber/ CEM CEM (CMI-7000T, Membranes International)	Vassilev et al., 2018
*S. ovata*	Acetate	Porous Ni-hollow fiber	IrO_2_-coated carbon cloth	2H_2_O → O_2_ + 4H ^+^+ 4e^–^	Two chamber/ Nafion 117	Bian et al., 2018
*A. woodii* *S. ovata* *M. maripaludis*	Acetate	CoP MoS_2_ NiMo	Platinized titanium mesh	2H_2_O → O_2_ + 4H ^+^+ 4e^–^	Two chamber/ Nafion 117	Kracke et al., 2019
Engineered *Clostridium ljungdahlii*	Acetate Butyrate Ethanol	Ni-P-modified carbon felt	A titanium mesh with iridium and ruthenium coating.	2H_2_O → O_2_ + 4H ^+^+ 4e^–^	Two chamber/ Nafion 117	Wang et al., 2020
Oxygen Adapted *S. ovata*	Acetate	Carbon felt	Carbon felt	2H_2_O → O_2_ + 4H ^+^+ 4e^–^	Two chamber/ Nafion 115	Shi et al., 2021
Anaerobic sludge	Methane	Carbon cloth	Carbon cloth coated with platinum powder	HS^–^ oxidized to SO_4_^2–^	Two chamber/ Nafion EC–NM–211	Dinh et al., 2022
*Acetobacterium* dominated mixed culture And pure culture of *Clostridium ljungdahlii*	Acetate	Graphite plate	Metal oxide coated titanium plate	2H_2_O → O_2_ + 4H ^+^+ 4e^–^	Two chamber/ Nafion 117	Roy et al., 2021
Enriched mixed culture	Butyrate	Carbon cloth connected to a stainless steel wire	Ti-MMO	2H_2_O → O_2_ + 4H ^+^+ 4e^–^	Two chamber/ Tubular cation exchange membrane CMI-1875Tl	Batlle-Vilanova et al., 2017
Enriched mixed culture	Ethanol, butyrate	Two pieces of graphite felts with a graphite rod sandwiched.	Titanium with an Iridium coated dimensionally stable anode (DSA)	2H_2_O → O_2_ + 4H ^+^+ 4e^–^	Two chamber/ 117 Nafion 117	Bajracharya et al., 2017
*Clostridium ljungdahlii*	Heptanoic acid, heptanol, caproate and hexanol	Round carbon cloth	Round stainless mesh plate	2H_2_O → O_2_ + 4H ^+^+ 4e^–^	Two chamber/ PEM PEM (No Specification)	Jabeen and Farooq, 2016
*Methanococcus maripaludis*	Methane	Graphite rod	Graphite rod	2H_2_O → O_2_ + 4H ^+^+ 4e^–^	Two chamber/ Nafion 117	Enzmann et al., 2019

Strategies to utilize CO_2_ as feedstock for the production of chemicals and fuels are of general high interest. Using CO_2_ shall allow creating sustainable carbon neutral (or even carbon negative) production routes and thus set the foundation for a circular biobased economy. CO_2_ is chemically stable; therefore, its utilization for chemical as well as (abiotic) electrochemical transformations requires large activation energy, respectively, overpotential (Gawel et al., 2022). In addition to chemical, biological synthesis from CO_2_ is also intensively researched, including photobiological synthesis or gas fermentation, which has been proven to yield multicarbon products from CO_2_ (Kondaveeti et al., 2020). These biological CO_2_ capturing methods possess benefits such as being based on widely available materials, ambient working conditions, being free of hazardous compounds, and being sustainable (Bajracharya et al., 2016a). In this line, MES that is using CO_2_ as carbon feedstock at cathodes was most deeply investigated. Hereby, electrons are supplied *via* a cathode to reduce carbon dioxide into products mainly by obligate anaerobic microorganisms that are capable of using the Wood-Ljungdahl CO_2_ fixation pathway (Nevin et al., 2011; Igarashi and Kato, 2017).

Among the most common products gained from CO_2_ *via* MES are acetate (CH_3_COOH) and ethanol (C_2_H_5_OH) (Table 1). *Sporomusa ovata*, *Sporomusa silvacetica*, *Sporomusa sphaeroides*, *Clostridium ljungdahlii*, *Clostridium aceticum*, and *Moorella thermoacetica* (Table 1) are among the autotrophic microorganisms that have been observed to generate acetate from CO_2_ through this pathway (Nevin et al., 2011). Another important group of autotrophic biocatalysts fixes CO_2_ *via* MES to methane, with the methanogens *Methanobacterium palustre* and *Methanococcus maripaludis* being most investigated (Enzmann et al., 2019). All these autotrophic biocatalysts or bioelectrocatalysts have in common that they are obligate anaerobic. Obligate anaerobes cannot sustain oxygen as they depend on low-potential flavoproteins for their respiration (Buckel and Thauer, 2018). Any exposure to oxygen can result in the biochemical and electrochemical formation of superoxide and hydrogen peroxide that are leading to oxidative stress and cell damage (Imlay, 2002). Thus, even small traces of oxygen in the MES setup can strongly hamper bioproduction. This means that most microorganisms suitable for performing MES from CO_2_ require (strictly) oxygen-free that is anoxic conditions to grow well and unravel their full performance.

To overcome or avoid this threat of oxygen toward the biocatalysts, many MES systems are operated with undefined mixed cultures at the biocathode (Gildemyn et al., 2015; Patil et al., 2015; Bajracharya et al., 2016b). However, a number of competitive microbial reactions happen in these MES, and therefore, the product (usually acetate) concentration profile fluctuates strongly. In a pure culture biocathode; in contrast, the production profile is more stable and reliable, indicating that no or only few competitive processes are present. In a direct comparison of pure and mixed culture MES, the highest production rate of a mixed culture biocathode was 1.35 mM/day, corresponding to 50% current efficiency, meanwhile, for a pure culture biocathode, the maximal production rate was 2.4 mM/day, corresponding to 89% current recovery between days 11 and 12 (Bajracharya et al., 2015). However, while the product spectrum was more stable for the pure culture, the overall operation was more unstable, likely due to the higher performance sensitivity of the pure culture catalyst, and this maximal performance was held only for 2 days of operation. Working with mixed cultures also greatly limits the perspectives to diversify the product spectrum since the community driving force is going toward thermodynamically stable products such as methane or acetate when methanogenesis is suppressed. The target production of diverse biochemicals or the application of molecular engineering for production pathways is not possible with mixed cultures.

The reactors used for MES are termed bioelectrochemical systems (BESs). A whole plethora of BES architecture is currently used when performing MES from CO_2_ (Table 1). Thereby, two fundamentally different types of reactors can be distinguished, namely, one-chambered and two-chambered BES. In one-chambered BES, anodic and cathodic reactions proceed in the same electrolyte solution that is the microbial medium, whereas in two-chambered reactors, cathode and anode are physically separated but ionically connected. It is commonly considered that in one-chambered reactors, compounds formed at one electrode may hamper the reaction at the other one and vice versa, whereas in two-chambered reactors, this is not the case, but resistance induced by the membrane separator may limit the overall performance (Krieg et al., 2018). For BES, very often reactor designs are used that are deduced from classical (abiotic) electrochemical cells. Furthermore, BESs are often not only inadequately described in terms of bioprocess engineering but they are also unsuitable for process development and scale-up (Rosa et al., 2019).

Bioelectrochemical systems for MES from CO_2_ using autotrophic microorganisms are commonly operated with the oxygen evolution reaction (OER) as counter-reaction at the anode (*vide infra*). Considering the strictly anaerobic nature of the discussed cathodic microbial biocatalysts, the evolved oxygen might more than likely hamper the MES performance.

## The challenges of designing bioelectrochemical systems for microbial electrosynthesis create a dilemma

Bioelectrochemical systems consist of an anode where oxidations take place and a cathode where reductions take place. In the case of two-chambered BES, a membrane is placed between the electrodes. When performing MES from CO_2_ at the cathode, the most common anode reaction is an electrolytic splitting of water by the OER (Eq. 1):


(1) $$2\,H_{2}\,O\to O_{2}+4\,H^{+}\,4\,e^{-}$$

**FIGURE 1:**
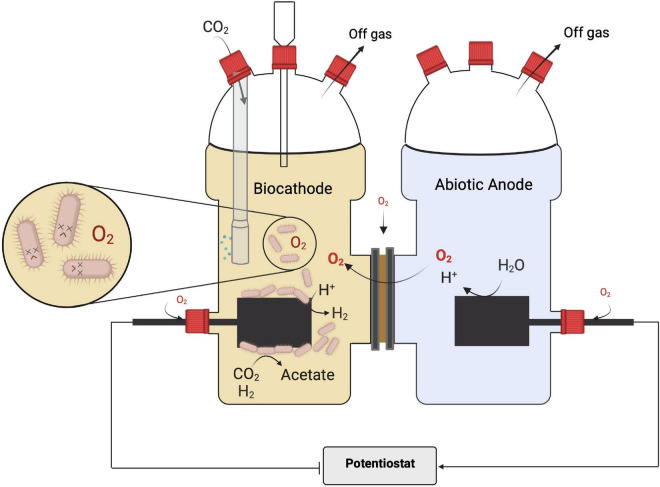
Microbial electrosynthesis in an H-type reactor as the archetype of a two-chambered bioelectrochemical system and often used for microbial electrosynthesis (MES) from CO_2_ (refer also to Table 1): the two chambers are separated by an ion-exchange membrane. As indicated, this setup provides more entry points for oxygen affecting the performance of the obligate anaerobic biocatalyst at the cathode with the main entry point being the membrane interface. Image created with BioRender.

The oxygen formed at the anode can penetrate into the cathode chamber. It can have a strong impact on the reactions in the BES, as recently illustrated for cathodic microbial electrochemical sulfate reduction (Dai et al., 2022). The presence of oxygen at the cathode is very intuitive for one-chamber BES. Yet, also in two-chamber BES, penetration of oxygen from the anode chamber cannot be completely circumvented with a separator like a cation or a proton exchange membrane (Harnisch and Schröder, 2009; Rahman et al., 2018). If oxygen from the anode as well as from the reactor environment is reaching the cathode (Figure 1), this will have several detrimental effects on the cathodic MES performance. First, and very importantly, oxygen can be reduced by abiotic or biotic cathode reactions (Eq. 2a) and thus decrease the coulombic efficiency (CE) (Dessì et al., 2021). The CE is the overall share of electrons being used for the target reaction, which in this study is the formation of products from CO_2_. Thus, any cathodic electron reacting directly or indirectly with oxygen is a lost electron in terms of production (Kocha et al., 2006). Second, as discussed above, oxygen is toxic for the most dominating MES biocatalysts and will reduce biocatalytic activity. Furthermore, the oxygen reduction reaction in addition to water can also yield hydrogen peroxide (Eq. 2b), which also leads to oxidative stress for the microorganisms (refer to above).


(2a) $$O_{2}+4\,H+4\,e^{-}\to 2\,H_{2}\,O$$

(2b) $$O_{2}+2\,H^{+}+2\,e^{-}\to 2\,H_{2}\,O_{2}$$

Thus, the oxygen evolution in multiple ways is directly affecting the performance of MES in terms of yields and rates. At the same time, it is without question that ionic contact between the anode and cathode chamber is required for operating any BES (Harnisch and Schröder, 2009). This contact has to be established with the high ionic conductivity of the separator at low resistance to the entire BES to limit energetic losses. Yet, separator materials providing high ionic conductivity usually also show a low resistance for penetration of oxygen: It is a dilemma!

## Ways out of the dilemma

It is a strong imperative to establish technical solutions for BES that shield the cathode compartment from O_2_ for successful MES. Different strategies that are briefly introduced below can help counteracting or resolving the above-described dilemma. In case the OER cannot be replaced as an anode reaction (refer to (b)), we proposed technical solutions to prevent or at least minimize O_2_ penetration to the cathode (refer to (c) to (g)) for lab-scale but also for scale-up applications.

### a) Use of microbial collaboration to scavenge away oxygen

In this study, the reported dilemma with oxygen toxification mainly applies to MES with defined microbial catalysts, especially pure cultures. However, many research groups successfully operated MES with undefined mixed cultures (Jourdin et al., 2014; Jourdin et al., 2015; Jiang et al., 2020). Using mixed cultures or reactor microbiomes intrinsically resolves the oxygen toxicity dilemma by immediate consumption of oxygen through aerobic microbial community members. While these systems are operating fairly robust, they create different limitations and challenges to upscaling MES. With undefined mixed cultures, highly efficient MES processes will stay limited to very few stable end-products such as methane, while other target products such as acetate or ethanol require a very good balance of the community activities to avoid their subsequent (re-consumption and conversion to unwanted side-products such as methane). Additionally, pure culture MES processes in the future might allow for the production of new, higher-value target compounds through genetic engineering of the biocatalysts, increasing the scope and impact of this technology. This important route of development is not at all available for mixed cultures. Most importantly, however, the oxygen dilemma regarding efficiency losses by oxygen reactions with cathodic components is just as pronounced for mixed cultures than for pure cultures. Thus, all the following strategies might also be suitable and advisable to improve the efficiency of MES with mixed microbial catalysts.

### b) Replacement of the anode reaction

The most effective strategy to avoid any harm of oxygen to the cathodic reaction certainly is its avoidance. An anode reaction that does not generate oxygen (Eq. 2a) or hydrogen peroxide (Eq 2b) is not affecting the cathodic microorganisms in general. On several anode materials, the OER (Eq.1) requires a remarkable overpotential. Therefore, oxidation of chemical compounds at lower overpotential in these materials seems very attractive, e.g., the oxidation of glycerol to formic acid (Eq. 3), or to glyceraldehyde (Eq. 4), or the oxidation of glucose to gluconic acid (Eq. 5). It was shown that this anode reaction can be more energetically and economically favorable than water oxidation (Jourdin and Burdyny, 2021).


(3) $$C_{3}\,H_{8}\,O_{3}+3\,H_{2}\,O\to 3\,C\,H_{2}\,O_{2}+8\,H^{+}+8\,e^{-}$$

(4) $$C_{3}\,H_{8}\,O_{3}\to C_{3}\,H_{6}\,O_{3}+2\,H^{+}+2\,e^{-}$$

(5) $$C_{6}\,H_{12}\,O_{6}+1\,H_{2}\,O\to C_{6}\,H_{12}\,O_{7}+2\,H^{+}+2\,e^{-}$$

Alternatively, chemical electron donors such as ferrocyanide (Yang et al., 2021) or anaerobic microbial oxidations could replace OER at the anode (Xiang et al., 2017). However, for the first, sustainable and complete recycling of the generated ferricyanide is currently not established and would lead to high economic and ecological costs. The latter approach comes with the intrinsic limitation that in this case also the anode potential needs to be carefully controlled to avoid toxic redox stress to the anodic microbial biofilm. As another option, Dinh et al. (2022) used the anodic oxidation of sulfide to sulfate as a counter-reaction for their cathodic electromethanogenesis.

### c) Purging inert gas in the cathode chamber

Purging inert gas such as nitrogen (N_2_) or CO_2_/N_2_ mixtures to the cathode chamber can be an option for small-scale laboratory experiments to assure anoxic conditions like it is used for other obligate anaerobic bioprocesses (Valdez-Vazquez et al., 2005). A continuous low stream of the CO_2_ feed supply might not be sufficient for oxygen removal, but higher gas fluxes come with some challenges. Already at the laboratory scale, we have to care about mechanical stress to microorganisms and in the case of pure N_2_, about removing the carbon source CO_2_ with the inert gas stream by outgassing. As water evaporation in the cathode has to be avoided, humidifying the gas is required at the lab scale (Wiegmann et al., 2018). Yet, purging high-volume streams of inert gas in the cathode chamber is no solution at technical scale and beyond. Apart from the environmental footprint that comes with using gaseous N_2_, the operational expenditures (opex) would be significant.

### d) Adapting the bioelectrochemical systems design

By changing reactor design, the share of O_2_ that is instantaneously removed from the anode solution can be decisively influenced. For example, a straightforward approach is the placement of the electrode. For instance, placing the anode on top of the cathode and in close vicinity to the water/gas interface will increase the mass transfer of O_2_ to the gas phase (Giddings et al., 2015). Thus, a lower amount of oxygen will penetrate into the anode chamber and thus less O_2_ is likely to penetrate the cathode. Thereby, it is important to keep a close distance between the cathode and anode. In the mentioned study, the electrode spacing was not changed. In any case, fluid dynamics and redox potential modeling approaches are recommended to obtain estimates on the impact of configurational changes on reactor performance (Rosa et al., 2019).

### e) Operating bioelectrochemical systems with oxygen scavengers

Using oxygen scavengers, i.e., materials that chemically bind or physically fix O_2_, seems highly promising but may come at higher costs for chemicals and at an additional overpotential. For instance, metal-organic frameworks (MOFs) could become of interest (DeCoste et al., 2014). Yet, the application of MOFs in aqueous solutions is challenging (Kavoosi et al., 2018), but tailoring the material may allow creating MOFs with specific properties needed for a certain MES. Thereby, a combination of improving the BES design and using a scavenger, for instance being placed close to the membrane, seems most promising. This means that the majority of oxygen would directly leave the anode solution, e.g., as bubbles, and only the O_2_ molecules close to the membrane would need to be scavenged away before penetrating to the cathode. For sustainability reasons, scavenger materials have to be recyclable for use in multiple MES operations.

### f) Using tailored membranes

As mentioned earlier, the membrane has to provide high ionic conductivity while minimizing O_2_ penetration. Therefore, tailoring membrane materials is required. Today, mainly polymer materials that are optimized for application in abiotic chemical reactors are used (Koók et al., 2019). In addition to these, ceramic membranes are promising, albeit they face the same dilemma of requiring to be repellant to oxygen at low ionic resistance. Apart from very few uses for fundamental research, due to the high resistance, the application of salt bridges for MES is not an option. Albeit having some application relevance, the use of advanced bipolar membranes currently does not seem to be a technical solution for scale-up, as well. Using bipolar membranes comes with increased opex and a loss of system efficiency, as the cell potential has to provide sufficient driving force for water splitting at the membrane (Harnisch and Schröder, 2009). Yet, using bipolar membranes can also come with a case-specific benefit of lowering the potential needed for the cathode reaction by a pH gradient across the membrane. This can partly outbalance the need for an external driving force for water splitting, which is, however, highly case-specific (Pärnamäe et al., 2021).

### g) Microbial strain adaptation to oxygen

A stepwise adaptive laboratory evolution (ALE) strategy can be used to increase the tolerance of anaerobic acetogens. Shi et al. (2021) developed two *Sporomusa ovata* strains that are adapted to 5% oxygen by a stepwise ALE. The adapted *S. ovata* strains showed better performance in autotrophic conditions in the presence of 5% oxygen where the OD reached 0.17 and 0.19 ± 0.02 for the adapted strains compared with the wild-type strain, which was not able to grow at all at this oxygen concentration. In addition, the adapted strains were 1.5-fold more active for acetate production in BES, which emphasizes the importance of solving the oxygen dilemma to overcome limitations.

As illustrated in this prospective study, there are several ways out of the oxygen dilemma for strict anaerobic MES. We are convinced that there is no silver bullet to solve this, and since there is no “one-fits-all” solution and MES concepts, target scales and economic boundaries will play great roles in choosing a certain route to mitigate the problem. All the above-mentioned strategies (Use of microbial collaboration to scavenge away oxygen) to (Microbial strain adaptation to oxygen) and even further options have to be considered, integrated, and investigated for finding solutions to enhance the function of pure culture MES. Independent of what the technical approaches are specifically, they need to be environmentally sustainable, biocompatible, feasible for scale-up, and economically sound. Intuitively, the option to avoid the problem by choosing alternative anodic reactions seems to be most appealing; however, if scalability or rate limitations greatly lack behind the anodic performance of OER, all the other options might be more feasible in the end. Therefore, only a very targeted and integrated solution will help to pave the way for defined MES from CO_2_ from the lab bench to biotechnological plants.

## Data availability statement

The original contributions presented in the study are included in the article/supplementary material, further inquiries can be directed to the corresponding author/s.

## Author contributions

MR and FH developed the concept and supervised MA and SA. MA and SA performed literature research, complied the table, designed the figure and wrote sections of the manuscript. FH wrote the first full draft of the manuscript. All authors contributed to manuscript revision, read, and approved the submitted version.
